# Supplementary material for: The Enigmatic Genetic Landscape of Hereditary Hearing Loss: A Multistep Diagnostic Strategy in the Italian Population
Source: Biomedicines. 2023 Feb 24;11(3):703. doi: 10.3390/biomedicines11030703 (PMC10045163; doi:10.3390/biomedicines11030703)
Supplement: Supplementary file 1 [file biomedicines-11-00703-s001.zip › biomedicines-2229916-supplementary.pdf]

**Table S1: Pathogenic variants within *USH2A* and *ADGRV1* genes identified in Italian cohorts.**
Data are aligned to the human genome reference build 37 (GRCh37). *USH2A* and *ADGRV1* gene variants were extracted considering a Minor Allele Frequency <0.05. Functional annotatation to determine the variant consequence was performed with bcftools' plug in "Split-VEP". Pathogenicity of the extracted variants was checked in HGMD®. Frequency of the extracted variants was checked in Non-Finnish Europeans in the GnomAD database. **A)** List of the extracted pathogenic variants within *USH2A* gene, **B)** List of the extracted pathogenic variants within *ADGRV1* gene.
Cohort = name of the considered Italian cohort (FVG = Friuli-Venezia Giulia, VBI = Val Borbera, CAR= Carlatino), Chr = chromosome, Pos = genomic position, rsID = unique identifier for a specific variant, Ref = reference allele, Alt = alternative allele, Freq = frequency of the reference allele, N carriers = number of pathogenic variant carriers, Freq gnomAD_NFE = frequency of the reference allele in Non-Finnish Europeans in the GnomAD database. NA= not available data. The values in brackets represent the total number of individuals for each cohort.

**A)**

**Frequency of *USH2A* pathogenic variants in Italian cohorts**

| Cohort | Chr | Pos | rsID | Ref allele | Alt allele | Freq | Consequence | HGMD *in-silico* pathogenicity prediction | N carriers | Freq gnomAD_ NFE |
|---|---|---|---|---|---|---|---|---|---|---|
| FVG (n=663) | 1 | 215628969 | rs111033402 | A | G | 0.0015083 | Missense | Damaging | 2 | 0.0004836 |
| | 1 | 215680331 | rs754979740 | T | C | 0.000754148 | Missense | Damaging | 1 | 0.00004418 |
| | 1 | 215728232 | rs111033364 | C | T | 0.000754148 | Stop gained | Damaging | 1 | 0.0001939 |
| | 1 | 215728281 | rs146264950 | C | T | 0.000754148 | Missense | Damaging | 1 | 0.0008272 |
| | 1 | 215879002 | rs111033533 | C | T | 0.000754148 | Missense | Damaging | 1 | 0.0006937 |
| | 1 | 216000489 | rs55958016 | C | T | 0.000754148 | Stop gained | Damaging | 1 | 0.00000000 |
| | 1 | 216078145 | rs886039867 | A | G | 0.00226244 | Missense | Damaging | 3 | NA |
| | 1 | 216289285 | rs146824138 | C | T | 0.0015083 | Missense | Damaging | 2 | 0.001109 |
| | 1 | 216325393 | rs780308389 | G | A | 0.000754148 | Missense | Damaging | 1 | 0.00001763 |
| | 1 | 216422149 | rs369806765 | C | T | 0.00678733 | Missense | Damaging | 9 | 0.000008816 |

| | | | | | | | | | | |
|---|---|---|---|---|---|---|---|---|---|---|
| VBI (n=424) | 1 | 216198494 | rs111033524 | C | A | 0.00117925 | Missense | Damaging | 1 | 0.0001412 |
| | 1 | 216247118 | rs80338902 | C | A | 0.00117925 | Missense | Damaging | 1 | 0.001405 |
| | 1 | 216325540 | rs371777049 | C | T | 0.00117925 | Missense | Damaging | 1 | 0.00006195 |
| CAR (n=124) | 1 | 215650687 | rs727504867 | G | A | 0.00806452 | Stop gained | Damaging | 2 | 0.00000000 |
| | 1 | 216073264 | rs111033409 | C | T | 0.00806452 | Missense | Damaging | 2 | 0.0001679 |
| | 1 | 216247118 | rs80338902 | C | A | 0.016129 | Missense | Damaging | 4 | 0.001405 |

**B)**

**Frequency of *ADGRV1* pathogenic variants in Italian cohorts**

| Cohort | Chr | Pos | rsID | Ref allele | Alt allele | Freq | Consequence | HGMD *in-silico* pathogenicity prediction | N carriers | Freq gnomAD_ NFE |
|---|---|---|---|---|---|---|---|---|---|---|
| FVG (n=663) | 5 | 90627592 | rs755371825 | C | A | 0.0015083 | Missense | Damaging | 2 | 0.00005314 |
| | 5 | 90684054 | rs41308846 | G | A | 0.0015083 | Missense | Damaging | 20 | 0.007248 |
| | 5 | 91153342 | rs41311625 | T | G | 0.000754148 | Missense | Damaging | 1 | 0.0004636 |
| VBI (n=424) | 5 | 90684054 | rs111033524 | G | A | 0.00235849 | Missense | Damaging | 2 | 0.007248 |
| | 5 | 90705414 | rs80338902 | G | A | 0.00117925 | Missense | Damaging | 1 | 0.00001779 |
| | 5 | 90763453 | rs371777049 | C | A | 0.00117925 | Missense | Damaging | 1 | 0.00559 |
| | 5 | 90848725 | rs201073459 | G | A | 0.00117925 | Missense | Damaging | 1 | 0.0001819 |
| CAR (n=124) | 5 | 90629222 | rs61744480 | A | C | 0.00806452 | Missense | Damaging | 2 | 0.002695 |
| | 5 | 90684054 | rs41308846 | G | A | 0.0120968 | Missense | Damaging | 1 | 0.007248 |
